# Causal Estimation of Long-term Intervention Cost-effectiveness Using Genetic Instrumental Variables: An Application to Cancer

**DOI:** 10.1177/0272989X241232607

**Published:** 2024-03-01

**Authors:** Padraig Dixon, Richard M. Martin, Sean Harrison

**Affiliations:** Nuffield Department of Primary Care Health Sciences, University of Oxford, Oxford, UK; MRC Integrative Epidemiology Unit, University of Bristol, Bristol, UK; MRC Integrative Epidemiology Unit, University of Bristol, Bristol, UK; Population Health Sciences, Bristol Medical School, University of Bristol, Bristol, UK; NIHR Bristol Biomedical Research Centre, University Hospitals Bristol and Weston NHS Foundation Trust and the University of Bristol, Bristol, UK; MRC Integrative Epidemiology Unit, University of Bristol, Bristol, UK; UK Health Security Agency

**Keywords:** genetics, cancer, instrumental variables, health care costs, cost-effectiveness, quality of life, Mendelian randomization

## Abstract

**Background:**

This article demonstrates a means of assessing long-term intervention cost-effectiveness in the absence of data from randomized controlled trials and without recourse to Markov simulation or similar types of cohort simulation.

**Methods:**

Using a Mendelian randomization study design, we developed causal estimates of the genetically predicted effect of bladder, breast, colorectal, lung, multiple myeloma, ovarian, prostate, and thyroid cancers on health care costs and quality-adjusted life-years (QALYs) using outcome data drawn from the UK Biobank cohort. We then used these estimates in a simulation model to estimate the cost-effectiveness of a hypothetical population-wide preventative intervention based on a repurposed class of antidiabetic drugs known as sodium-glucose cotransporter-2 (SGLT2) inhibitors very recently shown to reduce the odds of incident prostate cancer.

**Results:**

Genetic liability to prostate cancer and breast cancer had material causal impacts on either or both health care costs and QALYs. Mendelian randomization results for the less common cancers were associated with considerable uncertainty. SGLT2 inhibition was unlikely to be a cost-effective preventative intervention for prostate cancer, although this conclusion depended on the price at which these drugs would be offered for a novel anticancer indication.

**Implications:**

Our new causal estimates of cancer exposures on health economic outcomes may be used as inputs into decision-analytic models of cancer interventions such as screening programs or simulations of longer-term outcomes associated with therapies investigated in randomized controlled trials with short follow-ups. Our method allowed us to rapidly and efficiently estimate the cost-effectiveness of a hypothetical population-scale anticancer intervention to inform and complement other means of assessing long-term intervention value.

**Highlights:**

- The article demonstrates a novel method of assessing long-term intervention cost-effectiveness without relying on randomized controlled trials or cohort simulations.
- Mendelian randomization was used to estimate the causal effects of certain cancers on health care costs and quality-adjusted life-years (QALYs) using data from the UK Biobank cohort.
- Given causal data on the association of different cancer exposures on costs and QALYs, it was possible to simulate the cost-effectiveness of an anticancer intervention.
- Genetic liability to prostate cancer and breast cancer significantly affected health care costs and QALYs, but the hypothetical intervention using SGLT2 inhibitors for prostate cancer may not be cost-effective, depending on the drug’s price for the new anticancer indication. The methods we propose and implement can be used to efficiently estimate intervention cost-effectiveness and to inform decision making in all manner of preventative and therapeutic contexts.

This article implements methods for assessing the long-term intervention cost-effectiveness in the absence of data from randomized controlled trials (RCTs) and without recourse to Markov simulation or related types of cohort or individual-level simulations. We demonstrate this approach by estimating the cost-effectiveness of a novel population-wide anticancer prophylactic drug therapy repurposed from the treatment of diabetes. We proceed in 2 steps. We first used genetic instrumental variables using a Mendelian randomization study design^1,2^ to develop the estimates of the causal effect of liability or susceptibility to different site-specific cancers on, respectively, health care cost and quality-adjusted life-years (QALYs). We then used these estimates in a Monte Carlo simulation analysis to estimate directly the cost-effectiveness of a novel hypothetical anticancer intervention. These methods may be used to complement, prioritize, or inform the design of RCTs, cohort/individual-level disease simulation models, or other forms of long-term intervention evaluation.

Cancer, a major global cause of morbidity and death,^3–6^ is associated with important economic impacts on patients, carers, and health systems.^7–10^ Increases in the average age of populations, and improvements in the detection and treatment of cancer, are creating growing cohorts of individuals who have received cancer treatment, who are receiving treatment, or who may receive some form of cancer intervention for the rest of their lives.^8,11,12^ In turn, extended treatment modalities, new adjuvant regimens, and increases in the costs of therapies challenge the cost-effective delivery of care for cancer patients.^10,13–18^

Knowledge of how cancer status affects health economic outcomes such as health care cost and patient quality of life is central to providing cost-effective care to patients. Conventional study designs that examine associations between cancer status and these outcomes can be affected by measurement error and by omitted variable bias. Any traits, behaviors, or disease processes (including prodromal processes) that influence or are influenced by cancer status are likely in most cases to independently influence health care costs, quality of life, or both.

Our Mendelian randomization estimates are less likely to be biased by confounding from omitted variables and reverse causation than conventional analytic study designs, since these genetic variants are established at conception and should be unaffected by postconception confounding omitted variables such as behaviors (e.g., smoking), health status (e.g., comorbidities), or socioeconomic status (e.g., education level or occupation) that may jointly influence cancer status and these health economic outcomes.

We demonstrate the feasibility of directly estimating the health care costs and QALY impacts of cancer using robust causal methods and the use of these estimates to simulate intervention cost-effectiveness. The methods we implement can be used for other (noncancer) exposures, including disease, behavioral, and trait exposures, provided the genetic data necessary to implement robust Mendelian randomization analysis are available. There is increasing recognition that genetic data can support the selection and validation of drug targets.^19–22^ The methods we describe offer a further means of leveraging data on genetic variation to enhance the efficiency of intervention development and evaluation.

## Methods

We used Mendelian randomization to estimate the causal effect of cancer on health care costs and quality of life and, in turn, QALYs. Many introductions to Mendelian randomization are available.^1,23–25^ Briefly, Mendelian randomization relies on Mendel’s laws of inheritance, which describe how genetic variants are acquired by children from their parents. Some of these genetic variants transmitted in this manner are known to influence the risk of expressing specific phenotypes such as cancer. The allocation of variants from parents to children is random, conditional on the genotype of the parent.

At a population level, the association of genetic variants that influence the risk of, for example, a site-specific cancer permits the statistical identification of groups that differ in the levels of their exposure to the risk for that cancer. These genetic variants may be analyzed as instrumental variables. Their association with the studied exposure, ideally identified through genome-wide association studies, satisfies the first assumption of instrumental variable analysis: relevance. The second assumption of instrumental variables requires the instrument’s independence from all variables that confound the exposure-outcome relationship. The prenatal assignment of genetic variants minimizes postnatal influences, although this “independence” assumption cannot be fully tested. The third assumption, known as the exclusion restriction, requires that the instrument influences the outcome solely through the exposure of interest. Common genetic variants are likely to violate this assumption in 2 ways. First, linkage disequilibrium, reflecting correlation between genetic variants, may cause the violation if these variants affect the outcome independently of the exposure. Second, pleiotropy, in which a variant affects multiple phenotypes, can also breach the exclusion restriction if these additional phenotypes independently influence the outcome. We describe the steps we took to account for these assumptions in the “Data” section.

### Polygenic Risk Score Instrumental Variable Models

We developed polygenic risk scores (PRSs) for each cancer. These scores combine information from across the genome to indicate an individual’s genetic predisposition or liability to develop a particular trait, such as cancer.

The PRSs were created by obtaining the effect estimates on the risk of incident cancer from the genome-wide association studies (GWASs) described below in Box 1. These effect estimates were log-odds ratios specific to each cancer. We weighted each log-odds ratio by the precision with which it was estimated in the respective GWAS and then developed PRSs by applying these weighted effect estimates to information on the genetic variant carried (the “genetic dosage”) by each individual. The PRSs were used as instruments in “just-identified” 2-stage least square (2SLS) Mendelian randomization models. Just-identified models are instrumental variable models with as many instruments as exposures.^26,27^ We used the *ivreg2* package in Stata (version 17.0) with robust standard errors (SEs) to estimate these models.

**Box 1:** Creation of Cancer Polygenic Risk Scores^a^

Cancer	Subgroup	GWAS	SNPs	*R* ^2^
Bladder	All	Figueroa et al.^28^	10	0.57%
Breast	All	Michailidou et al.^29^	139	2.54%
	ER−		105	0.56%
	ER+		36	2.38%
Colorectal	All	Schumacher et al.^30^	8	0.30%
Lung	All	Wang et al.^31^	5	0.08%
	Adenocarcinoma		2	0.11%
	Squamous cell		4	0.13%
Multiple myeloma	All	Mitchell et al.^32^	13	0.72%
Ovarian	All	Phelan et al.^33^	12	0.20%
	Clear cell		1	0.01%
	Low malignant potential		4	0.10%
	Mucinous, invasive		3	0.002%
	Mucinous, invasive, low malignant potential		5	0.001%
	Mucinous, low malignant potential		4	0.002%
	Serous, high grade		15	0.24%
	Serous, high and low grade		15	0.25%
	Serous, low grade, low malignant potential		8	0.16%
	Serous, low malignant potential		3	0.10%
Prostate	All	Schumacher et al.^34^	123	4.04%
Thyroid	All	Gudmundsson et al.^35^	8	1.54%

ER−, estrogen receptor negative; ER+, estrogen receptor positive; GWAS, genome-wide association study; PRS, polygenic risk score; SNP, single nucleotide polymorphism.

a The “All” categories indicates main analyses, and other indicates additional analyses, using a GWAS for subgroups of cancer, although the exposure was all cancers in the respective category. *R*^2^ indicates the pseudo-*R*^2^ from a logistic regression of the cancer on its PRS, with no other included covariates.

In the first stage of the 2SLS model for each cancer, a cancer exposure variable was regressed on the PRSs with age at baseline assessment, sex, 40 genetic principal components (to control for the effects of ancestry-related population structure), and UK Biobank recruitment center as covariates. The predicted values of the coefficient on the instrument were then used in the second-stage regressions with either costs or quality of life as the outcomes. F-statistics from the first-stage regressions were inspected to assess instrument strength.^23^

Our Mendelian randomization analysis estimated the mean difference in the outcomes using an additive structural mean model,^24–26^ interpreted as the average change in each outcome caused by the hypothetical average effect of having the cancer versus not across the population.^36^ This assumes a constant effect of genetic susceptibility to each cancer on the cost and quality-of-life outcomes. An alternative assumption is to interpret the results as a local average treatment effect, which assumes a monotonic effect of genetic variants on each cancer exposure.^37^

### Multivariable Analyses

We compared the Mendelian randomization estimates with estimates from conventional separate multivariable linear regressions for QALYs and health care costs, with age, sex, 40 genetic principal components, and recruitment center as covariates. We performed Hausman endogeneity tests,^27^ in which a small *P* value indicates there was evidence the Mendelian randomization and multivariable effect estimates were different, although the power of these tests is relatively low.^38^

### Sensitivity Analyses

We conducted a variety of sensitivity analyses. We performed a multivariable adjusted analysis with additional variables (Townsend Deprivation Index, household income, highest educational qualification, ever smoked, and body mass index). Household income was self-reported according to 5 categories and top-coded for incomes greater than £100,000 per annum. The deprivation index was not self-reported, being based on local small-area deprivation estimates.

We reran the main Mendelian randomization analysis stratified by sex and age group (less than 50 y, 50–59 y, and 60+ y), both separately and together. The 60+ y category was chosen to reflect the age profile of participants in the UK Biobank cohort. We also stratified by when the participant was diagnosed with cancer, either before or after recruitment into UK Biobank, as participants who received their cancer diagnosis before recruitment were more likely to have lower health care costs associated with diagnosis and possibly curative treatment but more likely to have greater health care costs associated with managing progressing cancers and vice versa for participants who received their cancer diagnosis after recruitment.

We also examined potential violations of the exclusion restriction due to horizontal pleiotropy, which arises when one of the variants we include influences an outcome other than via the specific cancer exposure studied. To test for the presence of pleiotropy, we estimated overidentified Mendelian randomization models comprising the individual single nucleotide polymorphisms (SNPs) used in constructing cancer-specific PRSs. Heterogeneity in the effect of these SNPs on the cost and quality-of-life outcomes could indicate a violation of the exclusion restriction. These methods and their implementation are described in the Supplementary Material.

As a further sensitivity analysis, we estimated negative control models^39,40^ for sex-specific cancers that had the most robust evidence of causal effects in the Mendelian randomization analysis. We estimated sex-stratified linear regressions of each economic outcome against the breast cancer PRS (for men) and the prostate cancer PRS (for women) controlling for age, 40 genetic principal components, and indicators for UK Biobank recruitment center. We anticipated that we would observe effects consistent with the null for a prostate cancer PRS for women on these outcomes and likewise for a breast cancer PRS among men. We note that men may develop breast cancer^41^ but anticipated that the associations of breast cancer PRSs with costs and QALYs in men would differ substantially from the same associations among women. We further note that negative control tests of this type serve to complement conclusions drawn from the other types of inference but are not necessarily definitive on their own terms.

### Modeling the Cost-Effectiveness of a Novel Anticancer Intervention

We used our Mendelian randomization estimates of the causal impact of cancer on health care cost and QALYs to simulate the impact of a new population-wide anticancer intervention. Zheng et al.^42^ assessed the impact of sodium-glucose cotransporter 2 (SGLT2) inhibitors (a type of oral glucose-lowering drug) on prostate cancer risk. Their Mendelian randomization analysis found that SGLT2 inhibition, equivalent to a 1.09% reduction in HbA1c levels, lowered the odds of prostate cancer by 71% (odds ratio [OR] = 0.29, 95% confidence interval [CI]: 0.13–0.65) in their primary analysis and by 49% in replication analysis (OR = 0.51, 95% CI: 0.33–0.79).

In the absence of data on how such the hypothetical SGLT2 intervention would be dosed and costed for this new indication, we assumed that it would be offered in the form of 300 mg of canagliflozin, a particular type of SGLT2 inhibitor. As a monotherapy, a dose of 300 mg of canagliflozin had a broadly similar^43,44^ impact on HbA1c (approximately a 1% reduction) as did the Mendelian Randomization estimates in the Zheng et al.^42^ article. These comparisons are necessarily rather coarse since the Zheng et al.^42^ estimates relate to a largely unselected population, while the effectiveness estimates in the trials relate to effectiveness among diabetic patients, are dependent (to some degree) on baseline HbA1c levels, and we assume in our analysis no side effects from prophylactic treatment with these inhibitors.

We further note that the Zheng et al.^42^ estimates pertain to lifelong SGLT2 inhibition, and initiation of daily canagliflozin by, for example, the middle to early old age men represented in our UK Biobank cohort will likely understate the cost-effectiveness of this intervention. These are restrictive assumptions that may not apply to the use of these drugs in a “real-world” deployment but at least serve to indicate how these methodologies can enable an initial estimate of cost-effectiveness for a novel and otherwise untested intervention.

Our hypothetical intervention therefore assumed that every man in the analysis sample was offered and took daily SGLT2 inhibitors (and specifically 300 mg of canagliflozin) from their recruitment into the UK Biobank cohort. To estimate the causal impact of prostate cancer on health care cost and QALYs, we needed to estimate 3 quantities: 1) the proportion of men who developed prostate cancer, 2) the proportion of men who would not have developed prostate cancer had they received the intervention, and 3) the total health care and QALY cost saved by preventing those men from developing prostate cancer.

For the first quantity, we identified the proportion of all men in UK Biobank who were diagnosed with prostate cancer as well as within different age bands (less than 50 y, 50–59 y, and 60+ y), estimating the relevant SEs of each proportion:

$$\mathrm{SE}=\sqrt{\frac{\mathrm{proportion}\times (1-\mathrm{proportion})}{N}}$$

where *N* is the total number of men (overall or within each age band).

For the second quantity, we converted the ORs from Zheng et al.^42^ and the proportions estimated in quantity 1 into risk differences:

$$\mathrm{Risk}\;\mathrm{difference}\frac{n\times \mathrm{OR}}{\left(N-n+n\times \mathrm{OR}\right)}-\frac{n}{N}$$

where *n* is the number of men with prostate cancer, *N* is the total number of men, and OR is the odds ratio. For example, 6,155 of 144,032 men (4.3%) in UK Biobank were diagnosed with prostate cancer up to March 31, 2017, so an OR of 0.29 from Zheng et al.^42^ gives a risk difference of −3.0%.

For the third quantity, we used our multivariable adjusted or Mendelian randomization results estimates. We multiplied the risk difference (quantity 2) by the relevant multivariable adjusted or Mendelian randomization estimate (quantity 3) to estimate the annual total health care costs and QALYs saved by reducing incidence of prostate cancer with the intervention.

Because each quantity had associated uncertainty, we repeated the hypothetical intervention 10,000 times in a Monte Carlo analysis, with each draw taken from a normal distribution with a mean of the effect estimate and a standard deviation of the SE of the effect estimate. In this way, uncertainty in all relevant parameters was propagated through the simulation. The results of the simulation therefore reflect uncertainty around the effect estimates for cancer on the 2 outcomes. We present the median of the resulting estimates as the point estimate for the effect of the intervention, with the 2.5 and 97.5 percentiles as the 95% CI. This approach accounts for survival up to median follow-up.

We then compared the impact of SGLT2 inhibition on prostate cancer risk, assuming that the cost of doing so was equivalent to the cost of 300 mg of daily canagliflozin, to a “do-nothing” alternative, which reflects the absence of any accepted pharmacotherapeutic intervention for reducing population-wide prostate cancer risk. We compared the cost and effect of SGLT2 inhibition and a do-nothing comparator to a cost-effectiveness threshold of £20,000 using a net monetary benefit calculation. We adopted a health sector perspective for this cost-effectiveness analysis.

## Data

### Study Population

UK Biobank is a population-based health research resource consisting of more than 500,000 people who were recruited between the years 2006 and 2010 from 22 centers across the United Kingdom.^6^ We used medical data from hospital episode statistics (HES) linked to all participants up to March 31, 2015 (the censoring date of HES data in our analysis), and primary care (general practice) data linked to approximately 31% of UK Biobank participants registered with general practitioner surgeries using EMIS Health (EMIS Web) and TPP (SystmOne) software systems, up to March 31, 2017. The study design, participants, and quality control methods have been described in detail previously.^7–9^ UK Biobank received ethics approval from the Research Ethics Committee (REC reference for UK Biobank is 11/NW/0382).

We restricted the main analysis to unrelated individuals of White British ancestry (to avoid confounding by population stratification) living in England or Wales at recruitment. Related individuals were identified using kinship coefficients (measuring genetic relatedness) in the KING algorithm^45^ in a process described by Mitchell et al.^46^ In our sample, 38% of participants had primary care data.

### Identification of Cancers and Creation of PRSs

Following this process, the following cancers were available to be analyzed: bladder, breast, colorectal, lung, multiple myeloma, ovarian, prostate, and thyroid (Box 1). Supplementary Table S1 contains details for all SNPs used in these PRSs. We created PRSs for 8 cancers with an additional 13 PRS for subgroups of these cancers (2 for breast cancer, 2 for lung cancer, 9 for ovarian cancer). We implemented 2-sample Mendelian randomization^47,48^: the SNP-exposure associations were drawn from GWASs listed in Box 1, and the SNP-outcome associations were drawn from the UK Biobank data. To avoid biases from sample overlap,^49^ we ensured that these GWASs did not use data from the UK Biobank cohort.

We used age, sex, and UK Biobank recruitment center reported at the UK Biobank baseline assessment as covariables, as well as 40 genetic principal components derived by UK Biobank to control for population stratification.^43^

### Estimation of QALYs

We created health-related quality of life for all participants in UK Biobank from recruitment until March 2017. The details of how these data were constructed are given in Harrison et al.^50^ In brief, we used the data on quality of life in Sullivan et al.^51^ for 240 health conditions to create person-specific quality-of-life scores for every individual for all participants per day in UK Biobank from recruitment to March 31, 2017, or death, whichever came first. We multiplied the results for QALYs by 100 to give the percentage of a QALY changed by having versus not having the cancer. The QALY estimates represent the absolute percentage change in QALYs over an average year of follow-up; that is, they are not cumulative over time.

These health conditions were recorded in medical records or self-reported in the baseline UK Biobank recruitment questionnaire. These data were averaged over years of follow-up to create QALYs for each individual. See Supplementary Table S2 for the full list of health conditions used.

### Estimation of Health Care Costs

We have previously reported our approach to creating health care costs for the UK Biobank cohort.^50,52,53^ Briefly, we created health care costs from the records of procedure and diagnosis codes recorded in Hospital Episode Statistics linked to the UK Biobank cohort. This encompassed all inpatient care for publicly funded care in NHS hospitals from recruitment into UK Biobank to March 31, 2015. We used these data to calculate inpatient hospital costs per patient per year of follow-up. Primary care costs from recruitment into UK Biobank until March 31, 2017, were estimated from the costs of primary care consultations and from the cost of prescribed drugs (as of November 2019).

We combined annual primary care (consultation and prescription costs) and secondary care health care costs to create a total annual health care cost figure per person for each year of follow-up. We used multiple imputation by chained equations to impute primary care health care costs and other outcomes where data were missing. We created 100 imputed data sets^16^ and analyzed these data sets using Rubin’s rules. Other elements of care (e.g., private health care provided in private facilities) were not available.

The price for 300 mg of canagliflozin, used as an exemplar SGLT2 inhibitor, was taken from the 2019 NHS drug tariff price.^54^ The monthly price for 30 canagliflozin tablets of £39.20 was summed to give an annual cost figure of £470.

All costs in our analysis were expressed in 2019 prices. Cost data not expressed in 2019 prices were inflated to this price level using the NHS cost inflation index.^49^ Note that neither costs nor QALYs are discounted in our analysis. Instead, model coefficients represent change over an average year of follow-up and were estimated conditional on participant age in all cases.

### Data and Code Availability

The empirical data set will be archived with UK Biobank and made available to individuals who obtain the necessary permissions from the study’s data access committees. The code used to clean and analyze the data is available here: https://github.com/pdixon-econ/MR_cancer_simulation

## Results

We analyzed data on 310,913 unrelated individuals, of whom 166,981 (53.7%) were female (Table 1). The mean age at recruitment was 56.9 y (standard deviation: 8 y), and participants were followed up for a mean of 8.1 y (standard deviation 0.8 y). Median annual total health care costs per person were £601 (interquartile range: £212–£1,217). The most prevalent cancer we examined in UK Biobank was breast cancer (6.6% of women), followed by prostate cancer (4.3% of men), colorectal cancer (1.4%), bladder cancer (0.7%), lung cancer (0.7%), ovarian cancer (0.7% of women), multiple myeloma (0.2%), and thyroid cancer (0.2%).

**Table 1:** Summary of the Analysis Sample

Variable	All	Men	Women
*n*	310,913	144,032	166,881
Age at recruitment, years, $\bar{x}$ (*s*)	56.9 (7.99)	57.1 (8.10)	56.7 (7.90)
Years of follow-up, $\bar{x}$ (*s*)	8.1 (0.80)	8.1 (0.80)	8.1 (0.80)
Participants with complete primary care data, *n* (%)	96,331 (30.98)	44,671 (31.01)	51,660 (30.96)
Death before March 31, 2017, *n* (%)	10,519 (3.38)	6,447 (4.48)	4,072 (2.44)
Average QALYs per year (predicted), median (IQR)	0.78 (0.65 to 0.89)	0.78 (0.65 to 0.89)	0.78 (0.65 to 0.88)
Annual total health care costs, median (IQR)	£601 (£212 to £1,217)	£605 (£206 to £1,240)	£596 (£216 to £1,199)
Average primary health care costs per year, median (IQR)	£375 (£128 to £738)	£375 (£120 to £745)	£375 (£135 to £733)
Average secondary health care costs per year, median (IQR)	£88 (£0 to £494)	£90 (£0 to £502)	£87 (£0 to £487)
Breast cancer, *n* (%), female only			10,987 (6.58)
Bladder cancer, *n* (%)	2,096 (0.67)	1,592 (1.11)	504 (0.30)
Colorectal cancer, *n* (%)	4,473 (1.44)	2,575 (1.79)	1,898 (1.14)
Lung cancer, *n* (%)	2,065 (0.66)	1,126 (0.78)	939 (0.56)
Multiple myeloma, *n* (%)	541 (0.17)	319 (0.22)	222 (0.13)
Ovarian cancer, *n* (%), female only			1,114 (0.67)
Prostate cancer, *n* (%), male only		6,155 (4.27)	
Thyroid cancer, *n* (%)	453 (0.15)	112 (0.08)	341 (0.20)

IQR, interquartile range.

Results from imputed data, median, and IQR are the medians of the 100 imputed medians/IQRs.

### Results of Main Analysis

Estimates from conventional multivariable analysis indicated that cancer diagnoses reduced annual QALYs and increased annual health care costs per person (Table 2). The quantitative impacts were material for all cancers.

**Table 2:** Results from the Multivariable Adjusted Analyses

Exposure	Outcome	Effect Estimate (95% CI)
Bladder cancer	% Change in QALYs per year	−12.42% (−13.37% to −11.47%)
	Change in total health care costs per year	£1,346 (£1,282 to £1,410)
Breast cancer	% Change in QALYs per year	−4.73% (−5.14% to −4.31%)
	Change in total health care costs per year	£704 (£676 to £732)
Colorectal cancer	% Change QALYs per year	−11.20% (−11.87% to −10.53%)
	Change in total health care costs per year	£1,402 (£1,358 to £1,446)
Lung cancer	% Change in QALYs per year	−25.89% (−26.87% to −24.91%)
	Change in total health care costs per year	£1,564 (£1,499 to £1,629)
Multiple myeloma	% Change in QALYs per year	−18.06% (−19.90% to −16.22%)
	Change in total health care costs per year	£3,488 (£3,363 to £3,612)
Ovarian cancer	% Change in QALYs per year	−11.04% (−12.28% to −9.79%)
	Change in total health care costs per year	£1,431 (£1,347 to £1,516)
Prostate cancer	% Change in QALYs per year	−4.89% (−5.45% to −4.32%)
	Change in total health care costs per year	£552 (£512 to £592)
Thyroid cancer	% Change in QALYs per year	−5.20% (−7.19% to −3.22%)
	Change in total health care costs per year	£686 (£549 to £822)

CI, confidence interval; QALY, quality-adjusted life-year.

Table 3 presents the Mendelian randomization estimates for these exposures and outcomes. The estimates were relatively precise for breast cancer (£798 per year, 95% CI: £549–£1,048 and −5.51% QALYs per year, 95% CI: −9.32% to −1.70%) and for prostate cancer (£134 per year, 95% CI: −£217–£485 per year, and −2.68% QALYS per year, 95% CI: −7.48%–2.12%). Estimates were imprecise for all other cancers. The Mendelian randomization estimates are necessarily less precise than the corresponding conventional estimates, since their precision is a function of the amount of variance in each cancer exposure explained by the respective PRS.

**Table 3:** Results from the Mendelian Randomization Analyses

Exposure	Outcome	Effect Estimate (95% CI)
Bladder cancer	% Change in QALYs per year	1.83% (−42.23% to 45.88%)
	Change in total health care costs per year	£445 (£−2,569 to £3,459)
Breast cancer	% Change in QALYs per year	−5.51% (−9.32% to −1.70%)
	Change in total health care costs per year	£798 (£549 to £1,048)
Colorectal cancer	% Change in QALYs per year	−21.38% (−51.70% to 8.94%)
	Change in total health care costs per year	£1,917 (£−181 to £4,016)
Lung cancer	% Change in QALYs per year	−49.39% (−163.32% to 64.55%)
	Change in total health care costs per year	£2,732 (£−5,045 to £10,508)
Multiple myeloma	% Change in QALYs per year	−80.90% (−220.04% to 58.25%)
	Change in total health care costs per year	£10,720 (£1,308 to £20,133)
Ovarian cancer	% Change in QALYs per year	−100.16% (−213.00% to 12.68%)
	Change in total health care costs per year	£4,779 (£−2,289 to £11,847)
Prostate Cancer	% Change in QALYs per year	−2.68% (−7.48% to 2.12%)
	Change in total health care costs per year	£134 (£−217 to £485)
Thyroid cancer	% Change in QALYs per year	−94.41% (−207.80% to 18.98%)
	Change in total health care costs per year	£12,071 (£4,332 to £19,810)

CI, confidence interval; QALY, quality-adjusted life-year.

F-statistics were lowest for lung (F-statistic = 21) and ovarian (F-statistic = 25) cancers, suggesting possible weak instrument bias, but very high for prostate (F-statistic = 1,727) and breast (F-statistic = 1,824) cancers (see Supplementary Table S4). This suggests little evidence for weak instrument bias for breast and prostate cancers.

### Sensitivity Analysis

We found little evidence of pleiotropy in the Mendelian randomization estimates for breast and prostate cancer, suggesting that the exclusion restriction was probably not violated. There was little evidence of difference by sex for any cancer, although the most precise estimates were for breast and prostate cancer, which predominantly (breast cancer) or exclusively (prostate cancer) affect one sex. There was little difference in the effects on health care costs and QALYs per year when split by age. The negative control results were also consistent with the main results. The association with costs and QALYs of a prostate cancer PRS was consistent with the null among females, and the breast cancer PRS was consistent with the null amongst males. Full details of all sensitivity analyses are reported in the Supplementary Material.

### Evaluating the Impact of SGLT2 Inhibition on Prostate Cancer Risk

Under the assumptions of this analysis, the provision of SGLT2 inhibition as a prophylactic population-wide intervention dominates the “do nothing” status quo for men at risk of prostate cancer in the sense that it lowers health care costs and increases QALYs, but only if offered at zero cost (see Supplementary Table S6). This does not hold once the costs of the drugs themselves are accounted for (Table 4). As a sensitivity analysis, we also repeated this analysis assuming the more conservative OR (0.51, 95% CI: 0.33–0.79) of the replication analysis in Zheng et al. and report our findings in Supplementary Table S5.

**Table 4:** Results from the SGLT2 Intervention Analysis Compared with a “Do Nothing” Comparator

Age Group	Outcome	Analysis Method	Effect Estimate (95% CI)
All	Annual total health care costs	Mendelian randomization	£463.45 (£444.11 to £481.33)
		Multivariable adjusted	£451.47 (£446.71 to £460.80)
	QALYs per year	Mendelian randomization	0.13% (-0.1% to 0.4%)
		Multivariable adjusted	0.09% (0.04% to 0.13%)
	Net monetary benefit at a cost-effectiveness threshold of £20,000	Mendelian randomization	−£438 (−£494 to −£372)
		Multivariable adjusted	−£433 (−£452 to −£422)
<50 y	Annual total health care costs	Mendelian randomisation	£477.28 (£459.55 to £497.65)
		Multivariable adjusted	£468.75 (£467.81 to £469.55)
	QALYs per year	Mendelian randomisation	−0.06% (−0.28% to 0.15%)
		Multivariable adjusted	0% (−0.01% to 0.02%)
	Net monetary benefit at a cost-effectiveness threshold of £20,000	Mendelian randomisation	−£488 (−£545 to −£438)
		Multivariable adjusted	−£468 (−£470 to −£465)
50 to 59 y	Annual total health care costs	Mendelian randomisation	£468.29 (£446.21 to £490.54)
		Multivariable adjusted	£459.01 (£455.51 to £464.69)
	QALYs per year	Mendelian randomisation	0.02% (−0.3% to 0.37%)
		Multivariable adjusted	0.05% (0.02% to 0.09%)
	Net monetary benefit at a cost-effectiveness threshold of £20,000	Mendelian randomisation	−£462 (−£539 to −£385)
		Multivariable adjusted	−£448 (−£460 to −£439)
60+ y	Annual total health care costs	Mendelian randomisation	£448.79 (£414.55 to £478.39)
		Multivariable adjusted	£439.30 (£430.51 to £455.37)
	QALYs per year	Mendelian randomisation	0.33% (−0.07% to 0.81%)
		Multivariable adjusted	0.15% (0.07% to 0.22%)
	Net monetary benefit at a cost-effectiveness threshold of £20,000	Mendelian randomisation	−£384 (−£477 to −£268)
		Multivariable adjusted	−£409 (−£441 to −£388)

CI, confidence interval; QALY, quality-adjusted life-year.

Net monetary benefit becomes positive only if the SGLT2 inhibition drugs are offered at very modest prices; under the “All” scenario above, the drug becomes just cost-effective at an annual cost of approximately £32.

## Discussion

We developed the first Mendelian randomization estimates of the causal impact of cancer status on health care cost and on quality of life, the 2 outcomes necessary to undertake an economic evaluation of any cancer intervention. We studied the impacts of bladder, breast, colorectal, lung, multiple myeloma, ovarian, prostate, and thyroid cancers on these outcomes. The policy evaluation element of our article provides an example of how these types of estimates may be used to simulate the long-term impacts of anticancer intervention in the absence of, or in advance of, an RCT. These methods could also be used to prioritize future RCTs by identifying therapies most likely to be cost-effective.

Adjusted multivariable estimates indicated material impacts of cancer status on health care costs and quality of life for all 8 included cancers. Estimates from the Mendelian randomization models were much more imprecise, although genetic susceptibility for some cancers (especially breast and prostate cancers) was associated with reductions in QALYs and increases in health care cost. There was little evidence for heterogeneity and pleiotropy in violation of the exclusion restriction between SNPs for these cancers. Summary Mendelian randomization sensitivity estimates were similar to each other and broadly consistent with the main PRS estimates, indicating the same causal effect was likely being identified in each model without gross bias from violations of the exclusion restriction.

There was no apparent effect on our results when disaggregated by age (see Table S3 in the Supplementary Material). This tentatively indicates that the results apply to durations longer than the follow-up periods that we could study using available data sources. We suggest that this type of analysis be undertaken in all applications of this method to indicate whether the period over which the estimates are likely to apply. Markov cost-effectiveness models and similar simulations often adopt a lifetime perspective, whereas our method makes inferences that may be limited by the duration of follow-up available or the period for which effects are stable when disaggregated by age. This may not be an issue if the focus of an extrapolation or other assessment for which this and a Markov-type model is intended to examine pertains to short- or medium-term impacts.

The results from the Mendelian randomization analyses on cost and QALY outcomes are local average treatment effect estimates that represent the effect of lifelong exposure to higher germline genetic susceptibility to each respective cancer. The results of the analysis cannot be interpreted as, for example, the impact on costs of reducing cancer risk at any particular timepoint in the life course. Instead, they represent the impact on cost and QALY outcomes from changing long-term germline risk in this population for each specific cancer. They may not correspond to the magnitude of changes in these outcomes that would be observed through some other modification of cancer risk through, for example, manipulation of environmental influences on cancer risk.^55^

The cancer risks we modeled reflect the risks of incident disease. Our analysis includes SNPs that may influence both incidence and progression. For some cancers, especially prostate cancer, identifying aggressive cancer is arguably more important than understanding incidence since most tumors in (for example) the prostate do not influence overall survival.^56^ However, tumors that do not affect survival may still influence costs and quality of life, and their influence will be reflected in our estimates.

We did not capture all health care costs, including private care delivered in private health care facilities or outpatient care, neither of which were available for this cohort. This suggests our results may underestimate the costs associated with cancer.

Despite its size, UK Biobank is not representative of the UK population as its self-selected participants tend to be wealthier and healthier compared with the wider UK population.^39^ It is possible that our Mendelian randomization results underestimated the costs and quality-of-life impacts of cancer, since wealthier and healthier people may have more capacity to ameliorate the detrimental effects of increased genetic susceptibility to cancer. The impact of selection may have less significance for our conclusions^57,58^ than biases arising from violations of the instrumental variable assumptions for which we separately report sensitivity analyses and for more biologically distal exposures^59^ than we study. We controlled for genetic principal components in our analysis; there is evidence that this may not control for all remaining population or geographic structure,^41,60,61^ both of which could give rise to residual biases.

Parents may influence the environment in which their children are raised and could confound the SNP-cancer associations we estimate later in the life of these offspring. These impacts are likely to be modest given that costs and QALYs are measured in middle to early old age. Assortative mating refers to nonrandom mating; that is, individuals may preferentially mate (even if inadvertently) with others possessing variants that increases the risk of cancer. This may reintroduce environmental confounding otherwise avoided in Mendelian randomization.

Within-family Mendelian randomization studies can account for some of these biases. However, we did not have the statistical power to conduct these analyses since the binary cancer exposures we studied affected relatively few people in UK Biobank.^62^ In any event, there are no large cohorts of family trios that have reached middle and early old age that would have enabled this analysis for our UK Biobank cohort, and the magnitude of these biases may not be large for the cancer exposures that we study. For more visible traits such as body mass index^63,64^ and biologically distal phenotypes such as education,^65^ there is robust evidence of family-related effects,^66^ but it is less clear if these processes have any significance for our cancer exposures.

Among the conditions used to calculate QALYs are some (but not all) of the cancers that we also study as exposures (specifically bladder, breast, colon, lung, and prostate; see Supplementary Table S2). This creates a modest degree of circularity, but we do not consider this as problematic because the cancers we study contribute relatively little variance in QALYs across the full sample given their prevalence. We used this indirect method to calculate QALYs, since high-frequency longitudinal data on health-related quality of life and mortality outcomes are not typically collected in large cohorts with linked genetic information.

We estimated that a hypothetical intervention using repurposed SGLT2 inhibitors to prevent incident prostate cancer was unlikely to be cost-effective. However, we consider that these analyses demonstrate at least 2 important conclusions. The first is to lend support for the conduct of a RCT of SGLT2 inhibitors, given both the evidence from Zhang of their effectiveness and that the cost-effectiveness conclusions we present depend on the price at which SGLT2 inhibitors would be offered for a novel prostate cancer indication. The second conclusion is that the methods we have developed represent a feasible and rapid way of assessing the cost-effectiveness of both novel and established interventions.

We do not consider these methods as an alternative to RCTs, but they do offer promise as a means of efficiently prioritizing targets for investigation in RCTs, provided sufficient genetic evidence for the specific target of interest (see, e.g., Burgess et al.^20^) exists. They are likely to have particular value in triangulating evidence from different types of research design. The relatively low precision we observed in our estimates is partly a function of the type of exposure and of data sources used; in the Supplementary Material, we offer further reflections on how estimate precision might affect the wider use of these methods.

## Conclusion

Robust evidence of the causal impact of cancer exposures on health economic outcomes may be used as inputs into decision-analytic models of cancer interventions such as screening programs or simulations of longer-term outcomes associated with therapies investigated in RCTs with short follow-ups. We also demonstrated a method to estimate these parameters rapidly and efficiently to model the cost-effectiveness of a hypothetical population-scale anticancer intervention. These methods appear to be promising ways to inform and complement other means of estimating long-term intervention cost-effectiveness.

## Supplemental Material

sj-docx-1-mdm-10.1177_0272989X241232607 – Supplemental material for Causal Estimation of Long-term Intervention Cost-effectiveness Using Genetic Instrumental Variables: An Application to Cancer Supplemental material, sj-docx-1-mdm-10.1177_0272989X241232607 for Causal Estimation of Long-term Intervention Cost-effectiveness Using Genetic Instrumental Variables: An Application to Cancer by Padraig Dixon, Richard M. Martin and Sean Harrison in Medical Decision Making

sj-xlsx-2-mdm-10.1177_0272989X241232607 – Supplemental material for Causal Estimation of Long-term Intervention Cost-effectiveness Using Genetic Instrumental Variables: An Application to Cancer Supplemental material, sj-xlsx-2-mdm-10.1177_0272989X241232607 for Causal Estimation of Long-term Intervention Cost-effectiveness Using Genetic Instrumental Variables: An Application to Cancer by Padraig Dixon, Richard M. Martin and Sean Harrison in Medical Decision Making
