# Supplementary material for: Causal Estimation of Long-term Intervention Cost-effectiveness Using Genetic Instrumental Variables: An Application to Cancer
Source: Med Decis Making. 2024 Mar 1;44(3):283–95. doi: 10.1177/0272989X241232607 (PMC10988994; doi:10.1177/0272989X241232607)
Supplement: sj-docx-1-mdm-10.1177_0272989X241232607 – Supplemental material for Causal Estimation of Long-term Intervention Cost-effectiveness Using Genetic Instrumental Variables: An Application to Cancer [file sj-docx-1-mdm-10.1177_0272989X241232607.docx]

# Testing for the violations of exclusion restriction due to pleiotropy

Heterogeneity in effect estimates may indicate pleiotropy in violation of the exclusion restriction. Heterogeneity across the included single nucleotide polymorphisms (SNPs) can be assessed by comparing Cochran’s Q statistic $Q=\sum_{j=1}^{J} \frac{1}{\sigma_{Y_{j}}^{2}}{({\hat{\beta}_{j}-\hat{\beta}}_{IVW})}^{2}$ to the critical values of a chi-squared distribution. In this formula, there are *J* total SNPs, $\hat{\beta}_{IVW}$ is the inverse variance weighted (IVW) effect calculated for all *J* SNPs, $\hat{\beta}_{j}$ is the effect estimate for a specific SNP *j*, and $\sigma_{Y_{j}}^{2}$ the variance of the SNP-outcome association. The intuition for this formula is that any $\hat{\beta}_{j}$ with a large impact on the outcome that different from the overall IVW effect of all SNPs on the outcome may suggest multiple pleiotropic channels of influence of that SNP on the outcome not mediated by the specific cancer exposure investigated.

We used consensus methods and a modelling method (1) for our sensitivity analyses, each of which embodied different types of assumption about whether and in which respects the exclusion restriction may be violated. We first estimated the IVW estimates needed to calculate Cochran’s Q statistic, which involved calculating instrumental variable estimates for each SNP separately, and then combining them in random-effects meta-analysis with weights determined by the precision of the association between the SNP and the outcomes (healthcare costs and quality of life respectively). This assumes no violations of the exclusion restriction or no net effect of any such violations on the point estimates.

We then compared this restrictive baseline model against more models that are robust to different types of exclusion restriction violation. We implemented the Mendelian Randomization Egger estimator, which is consistent even if all SNPs are pleiotropic provided additional assumptions (relating to the association between instrument strength and the direct pleiotropic effect of SNPs) are met – see (2, 3). We also implemented a penalized weighted median estimator, which is consistent if at least 50% if the SNPs are valid instrumental variables (4). We also implemented a weighted mode estimator, which is consistent if the largest homogenous cluster of SNPs are valid even if more than 50% of SNPs are invalid as instrumental variables (5).

Note that results from these estimators are on a different scale to the instrumental variable estimates obtained from the polygenic risk score models. The over-identified sensitivity analyses reflect the measurement scales used for the outcomes in the respective source genome-wide association studies (GWASs), which are based on logistic regression. The scale of these outcomes therefore reflects a change in costs or quality of life per unit change in the log-odds of cancer status. These results are on a relative rather than absolute scale, reflecting the relative increase in genetic liability to cancer from increasing the number of risk-increasing alleles. By contrast, the effect estimates obtained from the 2SLS just-identified models are estimates of genetically influenced changes in disease status in the analysis population.

# Identification of cancers for inclusion in analysis sample and quality control of genetic data

We used UK Biobank as the source of our outcome data, the creation of which is described below. To avoid biases from sample overlap (6), we searched for genome-wide association studies (GWASs) on any type of cancer not using data from UK Biobank cohort. We searched the MR Base (mrbase.org) database of GWASs and the Elizabeth Blackwell Institute GWAS catalogue to identify cancers with at least clumped 2 single nucleotide polymorphisms (SNPs) associated with the cancer.

We also restricted our analysis to cancers with specific international classification of disease (ICD) codes at one decimal place, for example, multiple myeloma is coded as “230.0” in ICD-9, and “C90.0” in ICD-10. Where we found multiple GWASs for the same cancer, we preferentially used the GWAS with the most participants.

To account for linkage disequilibrium, we clumped the genome-wide significant SNPs for each cancer at an R^2^ threshold of 0.001 within a 10,000 kilobase window. We searched for proxies for all SNPs not in UK Biobank using the European subsample of 1,000 genomes as a reference panel (with a lower R^2^ limit of 0.6) (11). We used all included SNPs to construct PRS for each cancer, calculated as the weighted sum of the SNP effect alleles for all SNPs associated with each cancer, with each SNP weighted by the regression coefficient from the corresponding GWAS.

For some cancers, we found multiple GWASs which had subgroups of cancer as the outcome. In these cases, we created PRSs for the main cancer (e.g. breast cancer) as well as the available subgroups (e.g. ER+ and ER- breast cancers). There was insufficient detail in the ICD codes to determine the subgroup of cancer for all participants in UK Biobank, and as consequence the exposure used in the main analyses and reported below was for the general (e.g. breast cancer) exposure rather than its subtypes. In Supplementary Table S3 we also report Mendelian Randomization analysis using the available cancer subgroups.

# Results of Mendelian Randomization sensitivity analysis

The p-values for SNP heterogeneity were between 0.59 and 1.00 for Cochran’s Q statistic, and estimates from IVW, Egger, penalized weighted median, and weighted mode Mendelian Randomization estimates were all broadly similar, see Supplementary Table S3. There was little evidence of pleiotropy for the other exposures, although the number of SNPS available for analysis for these other cancers was smaller than for prostate and breast cancer.

We examined results stratified by sex (Supplementary Table S4). We also examined results split by both age and diagnosis time (pre- or post-baseline) see Supplementary Table S4. For breast cancer, the effect of breast cancer on QALYs per year was higher but more imprecise for both cancers diagnosed pre-baseline and post-baseline, whereas total healthcare costs per year were highest for cancers diagnosed post-baseline (Mendelian randomization estimate: £2,300 per year, 95% CI: £1,575 to £3,026). For prostate cancer, there was little difference in the effects on healthcare costs and QALYs per year when split by either diagnosis time or age.

The association with costs and QALYs of a prostate cancer PRS was consistent with the null amongst females (costs: -£10.04 (95% CI: -£20.97 to £0.88) and QALYs: 0.04% (95% CI: -0.13% to 0.21%)), and the breast cancer PRS was consistent with the null amongst males (costs: -£3.15 (95% CI: -£19.10 to £12.82) and QALYs: 0.03% (95% CI: -0.19% to 0.26%)). In each case, the coefficients represent the change in outcome for a unit increase in the PRS for each cancer. Full results from this analysis are reported in Supplementary material (Table S7).

# Precision and the wider use of these methods

The Mendelian Randomization estimates of disease exposure in relation to both costs and QALYs had wide confidence intervals. The precision of Mendelian Randomization estimates (as for other instrumental variable estimators) is a function of the variance explained by the instruments in the exposure variable. The proportion of variance explained for all manner of potential exposures will likely increase with further understanding of genetic contributions to disease, although in some cases (such as where heritability of the disease is low) the extent of future increases in variance explained may be limited.

Precision in our analysis was also influenced by the relatively low number of cancer cases observed in our outcome data set (UK Biobank), which happens to be relatively healthier than the wider general population from which the cohort was recruited. As follow-up continues in UK Biobank (and other cohorts), further disease cases will be identified, which will increase precision. Our exploration of these methods (7) for a continuous exposure (body mass index) were substantially more precise than our results for binary cancer exposures.

Note also that net benefit estimates depend on the price at which the SGLT2 inhibitor would be offered – Tables S5 and S6 in Supplementary Material demonstrate that the difference in cost between scenarios is largely driven by the cost of the drug, which we assumed would be equivalent to its list price for a diabetes indication. Assuming a smaller drug price, or studying an exposure with greater non-drug costs, would also have reduced the uncertainty around net benefit.

We demonstrated the feasibility of these methods for a binary exposure that had (on average) modest impacts on cost and QALYs and under conservative assumptions concerning the drug price of the intervention studied. Overall, we consider that the relatively low precision of some of our estimates need not be a barrier to the wider use of these methods, provided any results are interpreted with due consideration of the assumptions of this method and in the context of other available evidence.

References

1. Slob EAW, Burgess S. A comparison of robust Mendelian randomization methods using summary data. Genet Epidemiol. 2020;44(4):313-29.

2. Bowden J, Davey Smith G, Burgess S. Mendelian randomization with invalid instruments: effect estimation and bias detection through Egger regression. Int J Epidemiol. 2015;44(2):512-25.

3. Burgess S, Thompson SG. Interpreting findings from Mendelian randomization using the MR-Egger method. Eur J Epidemiol. 2017;32(5):377-89.

4. Bowden J, Davey Smith G, Haycock PC, Burgess S. Consistent Estimation in Mendelian Randomization with Some Invalid Instruments Using a Weighted Median Estimator. Genet Epidemiol. 2016;40(4):304-14.

5. Hartwig FP, Davey Smith G, Bowden J. Robust inference in summary data Mendelian randomization via the zero modal pleiotropy assumption. Int J Epidemiol. 2017;46(6):1985-98.

6. Burgess S, Davies NM, Thompson SG. Bias due to participant overlap in two-sample Mendelian randomization. Genet Epidemiol. 2016;40(7):597-608.

7. Harrison S, Dixon P, Jones HE, Davies AR, Howe LD, Davies NM. Long-term cost-effectiveness of interventions for obesity: A mendelian randomisation study. PLoS Med. 2021;18(8):e1003725.
